# Dynamics of the bacterial gut microbiota in preterm and term infants after intravenous amoxicillin/ceftazidime treatment

**DOI:** 10.1186/s12887-020-02067-z

**Published:** 2020-05-07

**Authors:** Romy D. Zwittink, Diny van Zoeren-Grobben, Ingrid B. Renes, Richard A. van Lingen, Obbe F. Norbruis, Rocio Martin, Liesbeth J. Groot Jebbink, Jan Knol, Clara Belzer

**Affiliations:** 1 Laboratory of Microbiology, Wageningen University, Stippeneng 4, 6708WE, Wageningen, The Netherlands; 2 Princess Amalia Dpt of Paediatrics, Dpt of Neonatology, Isala, Zwolle, The Netherlands; 3 Danone Nutricia Research, Utrecht, The Netherlands

**Keywords:** Gut microbiota, Preterm, Infant, Antibiotics, Next generation sequencing

## Abstract

**Background:**

It is important to understand the consequences of pre-emptive antibiotic treatment in neonates, as disturbances in microbiota development during this key developmental time window might affect early and later life health outcomes. Despite increasing knowledge regarding the detrimental effect of antibiotics on the gut microbiota, limited research focussed on antibiotic treatment duration. We determined the effect of short and long amoxicillin/ceftazidime administration on gut microbiota development during the immediate postnatal life of preterm and term infants.

**Methods:**

Faeces was collected from 63 (pre) term infants at postnatal weeks one, two, three, four and six. Infants received either no (control), short-term (ST) or long-term (LT) postpartum amoxicillin/ceftazidime treatment.

**Results:**

Compared to control infants, ST and LT infants’ microbiota contained significantly higher abundance of *Enterococcus* during the first two postnatal weeks at the expense of *Bifidobacterium* and *Streptococcus*. Short and long antibiotic treatment both allowed for microbiota restoration within the first six postnatal weeks. However, *Enterococcus* and *Bifidobacterium* abundances were affected in fewer ST than LT infants.

**Conclusions:**

Intravenous amoxicillin/ceftazidime administration affects intestinal microbiota composition by decreasing the relative abundance of *Escherichia-Shigella* and *Streptococcus,* while increasing the relative abundance of *Enterococcus* and *Lactobacillus* species during the first two postnatal weeks. Thriving of enterococci at the expense of bifidobacteria and streptococci should be considered as aspect of the cost-benefit determination for antibiotic prescription.

## Background

Neonatal infections are a major cause of mortality and morbidity, especially in preterm infants [1, 2]. Since symptoms of infection are mostly non-specific and infection can rapidly progress, most preterm infants are treated with broad-spectrum antibiotics before diagnosis. This, however, could result in overtreatment and increased risk of selection for resistant bacteria [3]. To reduce antibiotic use, the need for further antibiotic treatment is evaluated after 36–48 h. In addition to increasing the risk of antibiotic resistance, antibiotics might interfere with the development of the intestinal microbiota. During birth and thereafter, microbes rapidly colonise the human gastrointestinal tract. This process is not yet completely understood as it is highly dynamic and influenced by multiple host and environmental factors [4]. An abnormal pattern of bacterial colonisation has been observed in preterm infants compared to term infants, associated with greater exposure to factors like caesarean section, hospitalisation, formula feeding and antibiotic treatment [5, 6]. Gut microbiota development coincides with, and influences, development of the gastrointestinal tract and immune system. Disturbances in early life microbiota development could therefore affect early and later life health outcomes [7, 8]. Previous studies showed that the intestinal microbiota of preterm infants is affected by antibiotic treatment and characterised by high levels of facultative anaerobic bacteria and delayed colonisation with obligate anaerobes like *Bifidobacterium* [9–11]. The use of antibiotics in early-life, and corresponding disturbances of the gut microbiota, have been associated with negative health outcomes, including asthma, atopy and obesity [12–14]. Despite increasing knowledge about the effect of antibiotics on the microbiota, limited research focussed on antibiotic treatment duration [15–17]. We determined the effect of short-term and long-term postpartum antibiotic treatment on the gut microbiota throughout the first six postnatal weeks in 63 infants. Herein, the primary outcome was defined as the effect of antibiotic treatment duration on microbiota composition. As secondary outcome, the effect of other parameters on microbiota composition were studied, including gestational age, delivery mode, maternal antibiotics, enteral feeding tolerance, feeding type and respiratory support.

## Methods

### Subjects and sample collection

This study was part of an observational, single-centre, non-intervention study involving (pre) term infants admitted to the hospital level III neonatal intensive care unit or level II neonatal ward of Isala in Zwolle, The Netherlands. Infants born between 32 and 42 weeks gestation, admitted to the level II neonatal ward without major congenital malformation or malformations of the gastrointestinal tract, were eligible for inclusion. Informed consent was obtained from both parents of all individual participants. A total of 125 infants were eligible, of which seven infants were excluded due to incompliance, 40 infants were excluded due to incomplete longitudinal sampling or insufficient sample quantity, and 15 infants were previously included in a pilot study, resulting in 63 infants for inclusion. Infants were fed own mother’s milk, which was supplemented with (preterm) infant formula containing GOS/FOS when needed. Infants received either no (control, *n* = 28), short-term (< 3 days, ST, *n* = 22) or long-term (> 5 days, LT, *n* = 13) treatment with amoxicillin/ceftazidime (100 mg/kg/day amoxicillin / 100 mg/kg/day ceftazidime divided over two doses) during the first postnatal week. Antibiotic treatment started at the day of birth on the clinical suspicion of early-onset neonatal sepsis according to the hospital protocol (maternal risk factors as chorioamnionitis, fever, elevated infection parameters, Group B *Streptococcus*-carrier, preterm premature rupture of membranes < 35 weeks gestation, unexplained preterm birth with respiratory distress, clinical symptoms of sepsis or meningitis, need for artificial ventilation) and judgement by the attending physician. After 48 h, the need of antibiotic treatment was evaluated based on clinical signs, blood culture and serial C-reactive protein. Faecal samples were collected at postnatal weeks one, two, three, four and six, resulting in 263 samples, which were stored at − 20 °C until transfer to − 80 °C. Infant demographics are shown in Table 1.

**Table 1:** Patient demographics

		Control	ST	LT
Infants	**n**	28	22	13
	**Gestational age (weeks)**	34.8 ± 1.4	34.2 ± 2.2	37.2 ± 3.1
	**Birthweight (gram)**	2309 ± 456	2406 ± 588	3111 ± 863
	**Vaginal birth**	13 (46.4%)	15 (68.2%)	6 (46.2%)
	**Male**	13 (46.4%)	13 (59.1%)	8 (61.5%)
	**Preterm**	24 (85.7%)	19 (86.4%)	7 (53.8%)
	**Twin**	11 (39.3%)	5 (22.7%)	2 (15.4%)
	**AB treatment (days)**	0	2.2 ± 0.5	7.5 ± 2.2
	**CPAP**	5 (17.9%)	10 (45.5%)	0 (0%)
	**Food intolerant**	0 (0%)	0 (0%)	1 (7.7%)
	**TPN**	2 (7.1%)	0 (0%)	1 (7.7%)
	**Days until FEF**	7.0 ± 1.0	7.2 ± 1.3	7.6 ± 0.9
	**HM > 50% throughout 6 PNW**	19 (67.9%)	15 (68.2%)	7 (53.8%)
	**% HM throughout 6 PNW**	68 ± 27	71 ± 34	63 ± 32
	**Cause of infection:**			
	Proven sepsis			3 (23.1%)
	Clinical sepsis			6 (46.2%)
	Pneumonia			3 (23.1%)
	Meningitis			1 (7.7%)
	**Causative pathogen:**			
	Group B Streptococcus			4 (30.8%)
	*Escherichia coli*			1 (7.7%)
	Unknown			8 (61.5%)
Mothers	Preeclampsia	5 (17.9%)	3 (13.6%)	2 (15.4%)
	PROM	5 (17.9%)	5 (22.7%)	6 (46.2%)
	AB around birth	19 (67.9%)	15 (68.2%)	9 (69.2%)
	AB > 48 h after birth	2 (7.1%)	3 (13.6%)	4 (30.8%)

Abbreviations: AB: antibiotics, CPAP: continuous positive airway pressure, TPN: total parenteral nutrition, FEF: full enteral feeding, HM: human milk, PNW: postnatal week, PROM: prolonged rupture of membranes

### 16S rRNA gene amplicon sequencing

DNA extraction, library preparation and sequencing were performed by LifeSequencing S.L. (Valencia, Spain). DNA was extracted from 200 mg faeces using the QIAamp Fast DNA Stool Mini Kit (Qiagen), including cell disruption by bead beating. DNA was purified and concentrated using the PowerMag DNA clean-up kit (MoBio) and 50 ng of DNA was amplified according to the Metagenomic Sequencing Library Illumina 15,044,223 B protocol (Illumina) using 16S rRNA gene primers for region V3-V4 [18]. Libraries were quantified using the Quant-iT™ PicoGreen™ dsDNA Assay Kit (Thermofisher) and pooled prior to sequencing on the MiSeq platform (Illumina, 300 bases, paired-end).

### Data analysis

Read filtering, operational taxonomic unit (OTU)-picking and taxonomic assignment were performed using the NG-Tax pipeline with following settings: read length of 70, ratio OTU abundance of 2, classify ratio of 0.8, minimum percentage threshold of 0.5, identity level of 100%, error correction of 98.5, using the Silva 128 database [19, 20]. Coordinate analysis and differential abundance testing were performed in R (v3.6.1) using the packages phyloseq (v1.30.0), DESeq2 (v1.26.0), ggpubr (v0.2.4), microbiome (v1.8.0) and vegan (v2.5–6). To correlate the relative abundance of bacterial taxa with each other, Spearman’s rank correlation coefficient was determined. Prior differential abundance testing and correlation analysis, bacterial genera present in less than 25% of samples were removed to minimise zero-variance errors and spurious significance. For within infant (dependent) or between infants (independent) comparisons, the nonparametric Wilcoxon Signed Rank test and Kruskal-Wallis test were applied, respectively. To relate microbiota composition to clinical data, redundancy analysis (RDA) was performed using Canoco multivariate statistics software v5. Clinical factors included in the analysis are shown in Additional file 2 and were considered to have a significant influence on microbiota composition when the false discovery rate (FDR) corrected *p*-value was below 0.05.

## Results

### Differences in gut microbiota composition between infants receiving no, short or long antibiotic treatment

**Fig. 1:**
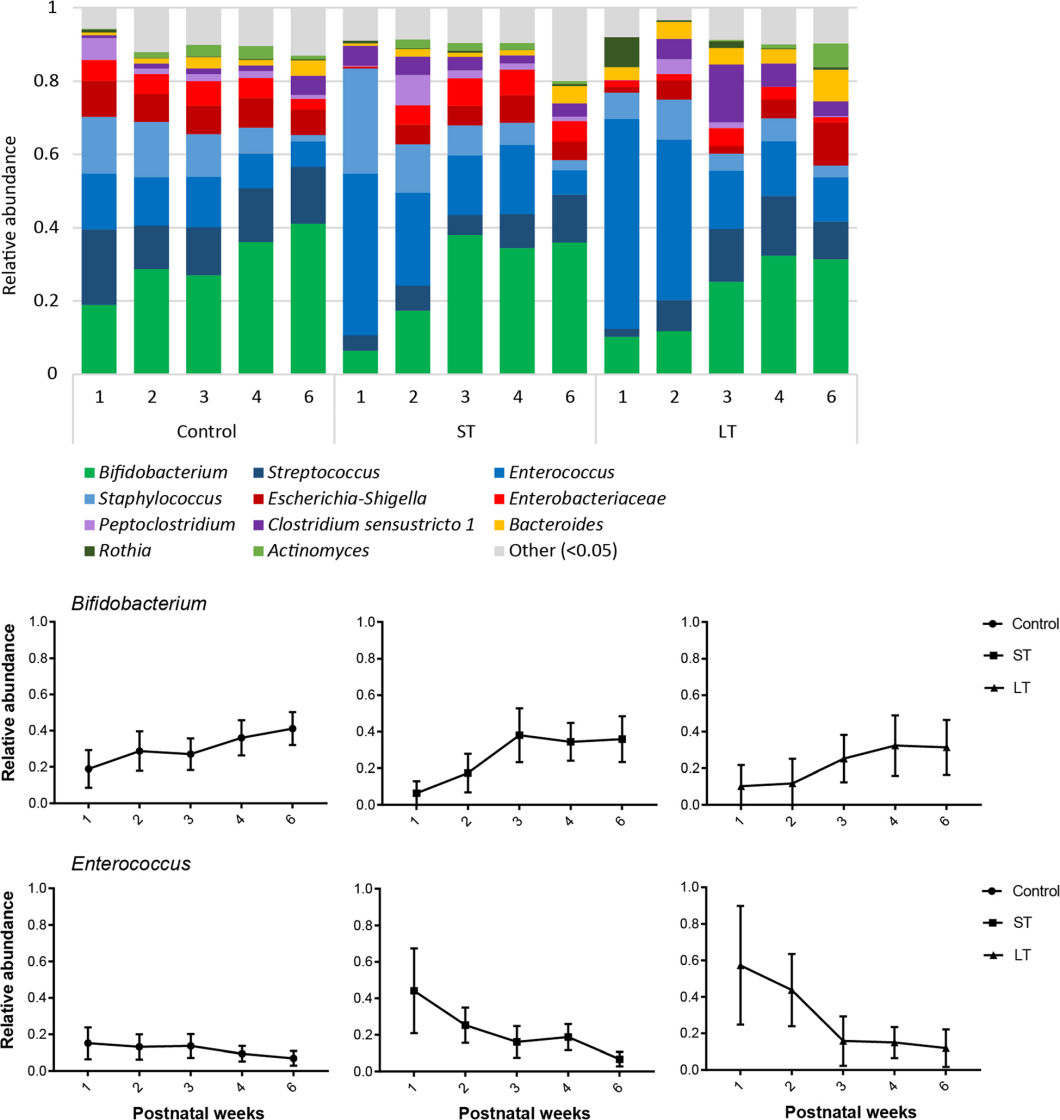
Microbiota composition profiles in control, ST and LT infants during the first six postnatal weeks, with a focus on *Bifidobacterium* and *Enterococcus*. Bar graphs: Average relative abundances per time point are shown. Line graphs: Mean ± 95% confidence interval are shown

Faecal microbiota composition was determined during the first six postnatal weeks in moderate- to late-preterm and term infants (32–42 weeks gestation) receiving either no (control), short-term (ST) or long-term (LT) antibiotic treatment during the first postnatal week (Fig. 1). Overall microbiota composition was significantly associated with antibiotic treatment duration during the first three postnatal weeks, but not at postnatal weeks four and six (Additional file 1). Differential abundance testing revealed that, at the first postnatal week, ST and LT infants’ microbiota contained significantly lower relative abundance of *Escherichia-Shigella* (Log2FoldChange = − 7.498, *p* = 0.0003; Log2FoldChange = − 5.442, *p* = 0.008, respectively) and *Streptococcus* (Log2FoldChange = − 4.011, *p* = 0.027; Log2FoldChange = − 3.795, *p* = 0.018, respectively), while higher abundance of *Lactobacillus* (Log2FoldChange = 27.979, *p* = 3.11 × 10^− 21^; Log2FoldChange = 6.743, *p* = 0.030, respectively) as compared to control infants. In LT infants, higher relative abundance of *Enterococcus* was observed at the second postnatal week, as compared to control infants (Log2FoldChange = 2.996, *p* = 0.005).

**Fig. 2:**
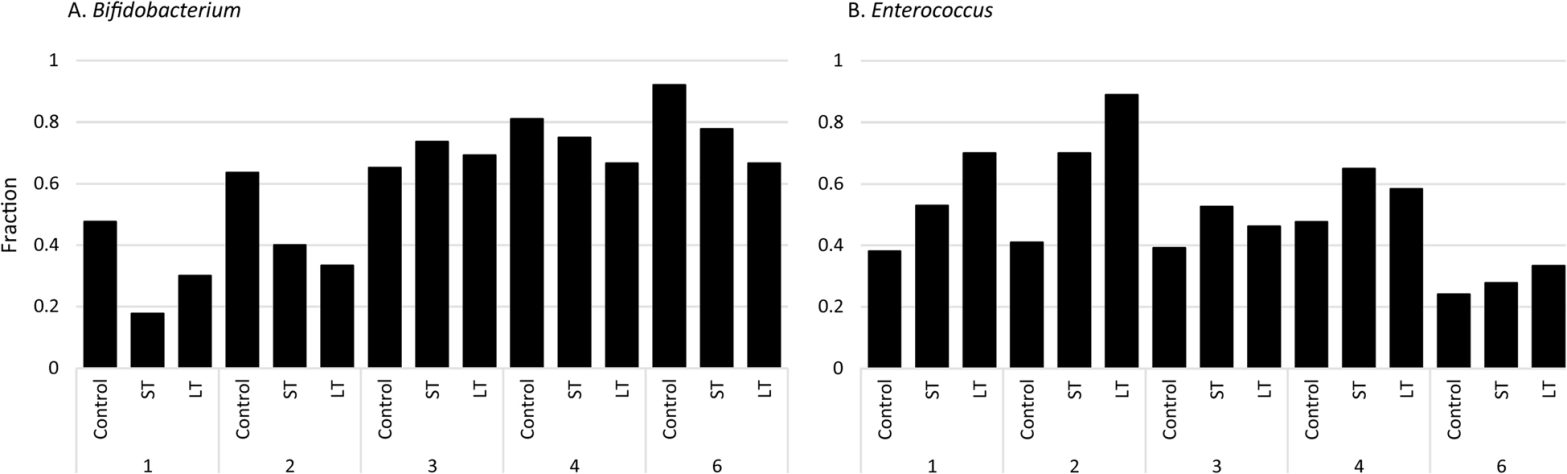
Fraction of infants in which *Bifidobacterium* (**a**) or *Enterococcus* (**b**) was an abundant member of the bacterial community. An abundant member was defined as a relative abundance of ≥10%

A comparison between ST and LT infants revealed significantly higher relative abundance of *Clostridium* sensu stricto 1 (Log2FoldChange = 21.783, *p* = 2.63 × 10^− 12^) and lower abundance of *Veillonella* (Log2FoldChange = − 26.954, *p* = 2.05 × 10^− 18^) at the first postnatal week. During the first two postnatal weeks, *Enterococcus* became an abundant member of the community in a higher percentage of LT than ST infants (Fig. 2). In addition, *Bifidobacterium* was an abundant community member in a higher percentage of ST than LT infants at postnatal weeks four and six (Fig. 2).

Community richness and diversity were not consistently affected by antibiotic treatment. Instead, community richness and diversity related to which taxa dominated the community. In mixed communities and communities in which *Bifidobacterium* was abundant, richness and diversity were higher than when either *Enterococcus*, *Streptococcus* or *Staphylococcus* was abundant, with lowest richness and diversity being observed for *Enterococcus* (Additional file 4).

### Gut microbiota succession in infants receiving no, short or long antibiotic treatment

The bacterial genera *Bifidobacterium, Streptococcus, Enterococcus, Staphylococcus, Escherichia-Shigella* and members of the *Enterobacteriaceae* family made up the biggest proportion of the (pre) term infant faecal microbiota (Fig. 1). Relative abundance of *Enterobacteriaceae* negatively correlated with abundance of *Escherichia-Shigella* (ρ = − 0.275, *p* = 4.0 × 10^− 6^). *Enterococcus* abundance negatively correlated with *Bifidobacterium* (ρ = − 0.260, *p* = 1.3 × 10^− 5^) and *Streptococcus* (ρ = − 0.279, *p* = 3 × 10^− 6^).

In control infants, the intestinal microbiota was characterised by high relative abundance of *Bifidobacterium*, *Streptococcus*, *Enterococcus*, *Staphylococcus*, *Escherichia*-*Shigella* and members of the *Enterobacteriaceae* family (Fig. 1). During the first six postnatal weeks, a trend of increasing relative abundance of *Lactobacillus* (Log2FoldChange = 5.075, *p* = 0.082) and decreasing *Staphylococcus* (Log2FoldChange = − 4.996, *p* = 0.084) was observed.

The intestinal microbiota of infants receiving short-term antibiotic treatment was characterised by high relative abundance of *Bifidobacterium*, *Enterococcus*, *Staphylococcus*, *Streptococcus*, *Escherichia*-*Shigella*, *Clostridium* and members of the *Enterobacteriaceae* family (Fig. 1). During the first six postnatal weeks, relative abundance of *Streptococcus* significantly increased (Log2FoldChange = 7.112, *p* = 0.015), and a trend of increasing relative abundance of *Bifidobacterium* (Log2FoldChange = 4.820, *p* = 0.098) was observed.

The intestinal microbiota of infants receiving long-term antibiotic treatment was characterised by high relative abundance of *Bifidobacterium, Enterococcus, Clostridium, Staphylococcus, Escherichia-Shigella, Bacteroides* and members of the *Enterobacteriaceae* family (Fig. 1). Over time, a trend of increasing *Streptococcus* (Log2FoldChange = 5.705, *p* = 0.050) was observed.

**Fig. 3:**
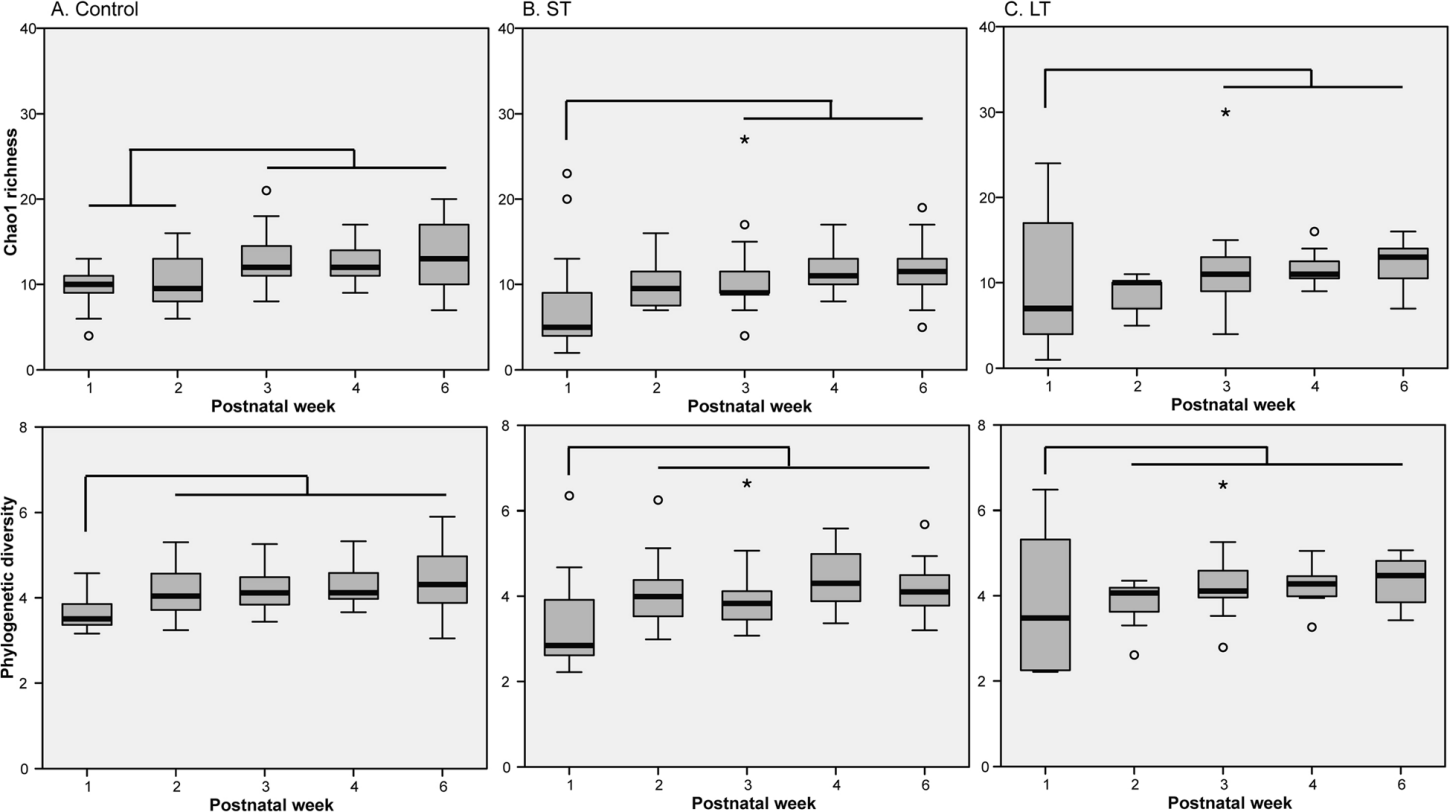
Community richness and diversity during the first six postnatal weeks in control (**a**), ST (**b**) and LT (**c**) infants. Boxplots show the median, 25th and 75th percentiles, and minimal and maximal values with the exception of outliers (circles) and extremes (asterisks). Differences in richness and diversity between time points were determined using the Wilcoxon Signed Rank test with Monte Carlo permutation

Bacterial richness and diversity generally increased over time in all infants, independent of antibiotic treatment duration (Fig. 3).

### Clinical variables associated with microbiota composition during the first six postnatal weeks

The effect of various clinical characteristics on microbiota composition was determined by redundancy analysis. Clinical variables explaining variation in microbiota composition were group-specific, and included delivery mode, feeding-related factors and postnatal age (Table 2). When combining the three groups, antibiotic treatment duration explained 2.5% of variation in microbiota composition between samples (Table 2). In addition, postnatal age (5.5%), gender (2.9%), days until full enteral feeding (2.2%), delivery mode (2.0%) and gestational age (1.5%) affected microbiota composition (Table 2). Males were associated with increased abundance of *Escherichia-Shigella*, while females were associated with increased abundance of other members of the *Enterobacteriaceae* family (Additional file 3). This difference was statistically significant at postnatal weeks two, three and four (*p* < 0.05). Increased postnatal age, no antibiotic treatment, less days until full enteral feeding and higher gestational age were associated with increased abundance of *Bifidobacterium* (Additional file 3). Regarding gestational age, this study comprised preterm and term infants. The intestinal microbiota of term and preterm infants responded to antibiotic treatment in a similar manner (Additional file 5). On average, *Bifidobacterium* abundance was higher in term compared to preterm infants, however, this was not statistically significant.

**Table 2:** Factors explaining the variation in microbiota composition between samples as determined by redundancy analysis. Factors significantly explaining the variation are shown

Control			ST			LT			ALL		
Factor	Explains (%)	FDR p-value	Factor	Explains (%)	FDR p-value	Factor	Explains (%)	FDR p-value	Factor	Explains (%)	FDR p-value
Vaginal delivery	7.7	0.00433	Postnatal age	7.4	0.01533	Postnatal age	7.8	0.016	Postnatal age	5.5	0.006
Postnatal age	5.5	0.00433	PROM No	5.4	0.01533	Vaginal delivery	6.9	0.016	AB1 duration	2.5	0.006
Primary c-section	3.2	0.00433	PROM Yes	5.4	0.01533	TPN (days)	6.5	0.016	Female	2.9	0.006
Secondary c-section	3.2	0.00433	Male	4.9	0.01533				Male	2.9	0.006
Days until FEF	3.0	0.00433	Female	4.9	0.01533				Days until FEF	2.2	0.006
HM_6weeks (%)	2.9	0.00433							Primary c-section	2.0	0.006
Female	2.7	0.00433							GA (weeks)	1.5	0.006
Male	2.7	0.00433									
CPAP Yes	2.3	0.00743									
CPAP No	2.3	0.00743									
No Maternal AB	1.6	0.02022									
Maternal AB	1.6	0.02022									
GA (weeks)	1.6	0.02022									

Abbreviations: FDR: false discovery rate, C-section: caesarean section, FEF: full enteral feeding, HM: human milk, CPAP: continuous positive airway pressure, AB: antibiotics, GA: gestational age, PROM: prolonged rupture of membranes, TPN: total parenteral nutrition

## Discussion

Intravenous antibiotic administration for prevention and treatment of infection and sepsis occurs frequently in neonatal units. Therefore, it is of great relevance to study side effects of antibiotic treatment, including its effect on gut microbiota development. We studied the effect of postpartum antibiotic treatment duration on microbiota development in 63 (pre) term infants during the first six postnatal weeks.

The genera *Bifidobacterium, Streptococcus, Enterococcus, Staphylococcus, Escherichia-Shigella* and members of the *Enterobacteriaceae* family made up the biggest proportion of the (pre) term infant faecal microbiota. Relative abundances of *Enterococcus* and *Staphylococcus* decreased during the first six postnatal weeks, while abundances of *Streptococcus* and *Bifidobacterium* increased. Overall microbiota composition was associated with antibiotic treatment duration during the first three postnatal weeks. Short- and long-term antibiotic treatment with amoxicillin/ceftazidime affected microbiota composition by decreasing the relative abundance of *Escherichia-Shigella* and *Streptococcus*, while increasing the relative abundance of *Enterococcus* and *Lactobacillus* species, which is in concordance with our previous findings [17]. Ceftazidime and amoxicillin are broad-spectrum β-lactam antibiotics, targeting Gram-positive and -negative bacteria. It has been shown that *Bifidobacterium* species are sensitive to β-lactam antibiotics and that treatment with amoxicillin can greatly influence the composition of *Bifidobacterium* species in infant intestinal microbiota [21–23]. As such, establishment of a *Bifidobacterium*-dominated microbiota can be delayed or prohibited. Strikingly, relative abundance of *Enterococcus* increased after long-term antibiotic treatment, which might indicate antibiotic resistance, as it is a target organism of amoxicillin. As some *Enterococcus* species emerged from gut commensals to nosocomial pathogens, this might pose a health risk for the infants [24]. Indeed, *Enterococcus* species have been identified as causative organism in late-onset sepsis [25, 26]. However, the applied methodology herein cannot reliably identify *Enterococcus* to species level, and whether absolute abundances of *Enterococcus* increased has not been elucidated in the study herein.

Few differences in microbiota composition were observed between infants receiving short- or long-term antibiotic treatment. However, its noteworthy that *Enterococcus* became an abundant community member in a higher percentage of long- than short-treated infants. As well, *Bifidobacterium* did not become an abundant community member in a higher percentage of long- than short-treated infants. This indicates that long antibiotic treatment has a more profound effect on microbiota development than short treatment, similar to what we have previously reported [17]. High inter-individual variation, in combination with the relatively small number of long-treated infants, most certainly decreased statistical power. In addition, inclusion of all infants, instead of studying a carefully selected subset, did not allow to prevent possible bias by parameters like gestational age, delivery mode, gender, maternal antibiotics and feeding. For example, 46.2% of long-treated infants were born at term, while only 13.6% of short-treated infants were born at term. Nevertheless, stratification of infants based on preterm and term birth indicated similar response to antibiotic treatment.

Community richness and diversity were not consistently affected by antibiotic treatment. Instead, richness and diversity increased over time, and were related to which bacterial taxon was abundant. Richness and diversity were lower when either *Enterococcus*, *Streptococcus* or *Staphylococcus* was abundant, and higher when other bacterial taxa, including *Bifidobacterium,* were abundant. As the relative abundance of *Bifidobacterium* increased over time, postnatal age and *Bifidobacterium* abundance were related, hindering the elucidation of their sole effect on community richness and diversity.

In addition to antibiotic treatment duration, microbiota composition was associated with postnatal age, gender, days until full enteral feeding, delivery mode and gestational age. Increased gestational and postnatal age and less days until full enteral feeding were associated with higher abundance of early life coloniser *Bifidobacterium*. A *Bifidobacterium*-dominated microbiota is more representative of microbiota development in term, vaginally born, breast-fed infants, which is considered most beneficial during early life development [27]. The beneficial effect of *Bifidobacterium* species is speculated to be obtained by providing protection against pathogens and via its immune modulating properties [28]. Since dominance by *Bifidobacterium*, compared to other bacterial taxa, allowed for higher community richness and diversity, we speculate that *Bifidobacterium* species control, but not outcompete, other bacterial species. Bifidobacteria could therefore play an important role in development of a healthy and diverse ecosystem that promotes tolerance induction and immune system maturation. In addition, bifidobacteria are optimal milk degraders, and known for their role in degradation of simple and complex sugars like human milk oligosaccharides [29]. Early differences in microbiota composition may therefore affect an infants’ food digestion capacity and subsequent energy harvest [30, 31]. This is particularly relevant for preterm born infants with protein and energy deficits [32, 33]. Regarding gender, males microbiota contained higher abundance of *Escherichia-Shigella,* while the microbiota of females contained more members of the *Enterobacteriaceae* family that could not be classified to genus level. Several studies have shown that gut microbiota composition differs between adult males and females [34, 35], but gender-effect during early life is relatively unexplored [36]. It is important to note that while many clinical variables were included in the analysis, they did not capture the full extent of microbiota variation observed between samples. As such, unknown determinants affecting microbiota composition in (pre) term infants remain.

## Conclusions

Our findings show that intravenous administration of amoxicillin/ceftazidime affects intestinal microbiota composition, particularly by decreasing the relative abundance of *Escherichia-Shigella* and *Streptococcus,* while increasing the relative abundance of *Lactobacillus and Enterococcus* species during the first two postnatal weeks. Short and long antibiotic treatment both allow for intestinal microbiota restoration within the first six postnatal weeks as characterised by increasing relative abundance of *Bifidobacterium* species. Long treatment, however, potentially has more enduring effect on microbiota development than short treatment, but this needs to be further elucidated. Although being of short-term, the rise of enterococci at expense of bifidobacteria and streptococci, including the potential effect of disturbed microbiota development on health outcomes, should be considered as aspect of the cost-benefit determination for antibiotic prescription.

## Supplementary information

**Additional file 1.** Principal Coordinate Analysis (PCoA) plots using Bray-Curtis distances. The association of microbiota composition with postnatal week (WoL) or antibiotic treatment duration (AB1_dur) was assessed by Permutational multivariate analysis of variance (PERMANOVA) using the ‘adonis’ function with 999 permutations

**Additional file 2.** Clinical factors included during redundancy analysis (RDA)

**Additional file 3.** Redundancy analysis using microbiota composition profiles from (A) control infants and (B) all infants. Species with a 15-100% fit into the ordination space and explanatory variables that significantly explain variation are shown. Abbreviations: C-section: caesarean section, AB: antibiotics, FEF: full enteral feeding, PNW: postnatal weeks.

**Additional file 4.** Richness and diversity in samples with different dominating bacterial taxa. Taxa were considered dominant in a sample when it was the most abundant taxon and at least 10% more abundant than the second most abundant taxon. When the difference between the two most abundant taxa was less than 10%, is was considered a mixed community. Boxplots show the median, 25th and 75th percentiles, and minimal and maximal values with the exception of outliers (circles) and extremes (asterisks). Lines above the graph indicate between which communities a significant difference in richness/diversity was observed

**Additional file 5..** Microbiota composition profiles during the first six postnatal weeks in preterm and term infants receiving no, short or long antibiotic treatment. Average relative abundances per time point are shown.

## Data Availability

Raw sequencing data and supporting metadata are available in the European Nucleotide Archive (http://www.ebi.ac.uk/ena) under study accession PRJEB26802.

## Abbreviations

FDR: False discovery rate
OTU: Operational taxonomic unit
RDA: Redundancy analysis
ST: Short-term
LT: Long-term
