# Supplementary material for: Dynamics of the bacterial gut microbiota in preterm and term infants after intravenous amoxicillin/ceftazidime treatment
Source: BMC Pediatr. 2020 May 7;20:195. doi: 10.1186/s12887-020-02067-z (PMC7204001; doi:10.1186/s12887-020-02067-z)
Supplement: Supplementary file 1 [file 12887_2020_2067_MOESM1_ESM.docx]

Additional file 1. **Principal Coordinate Analysis (PCoA) plots using Bray-Curtis distances.** The association of microbiota composition with postnatal week (WoL) or antibiotic treatment duration (AB1_dur) was assessed by Permutational multivariate analysis of variance (PERMANOVA) using the 'adonis' function with 999 permutations.

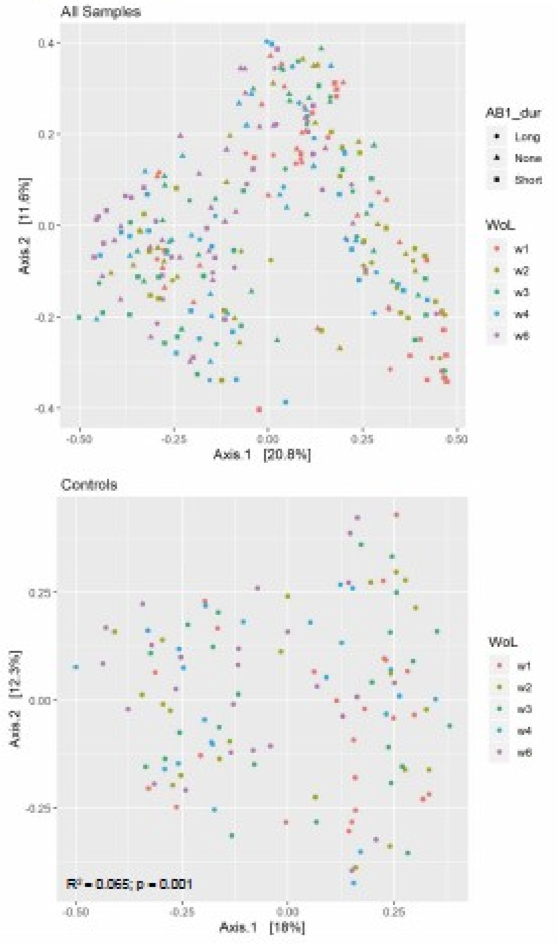

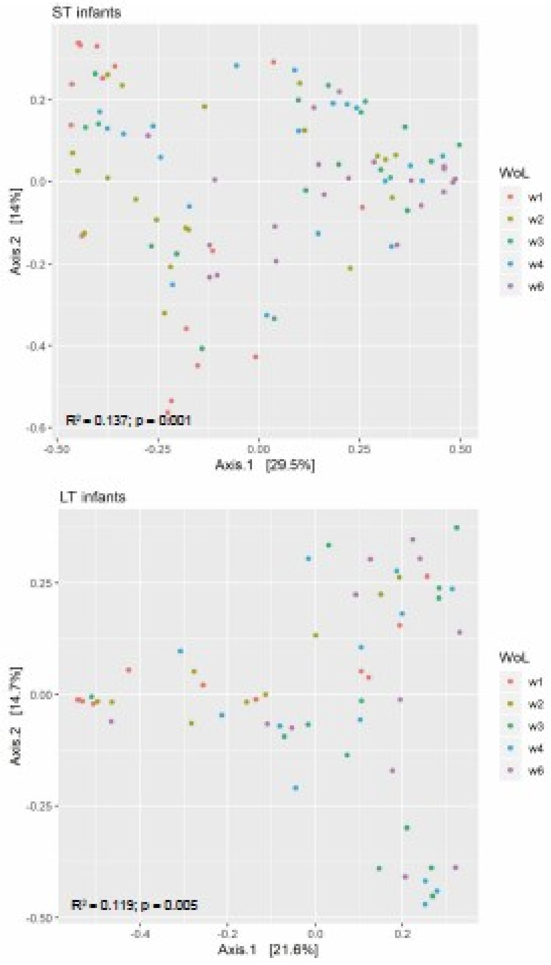
ST infants
Axis.2 [14%]
Axis.1 [29.5%]
R² = 0.137; p = 0.001
WoL
w1
w2
w3
w4
w6
LT infants
Axis.2 [14.7%]
Axis.1 [21.6%]
R² = 0.119; p = 0.005
WoL
w1
w2
w3
w4
w6

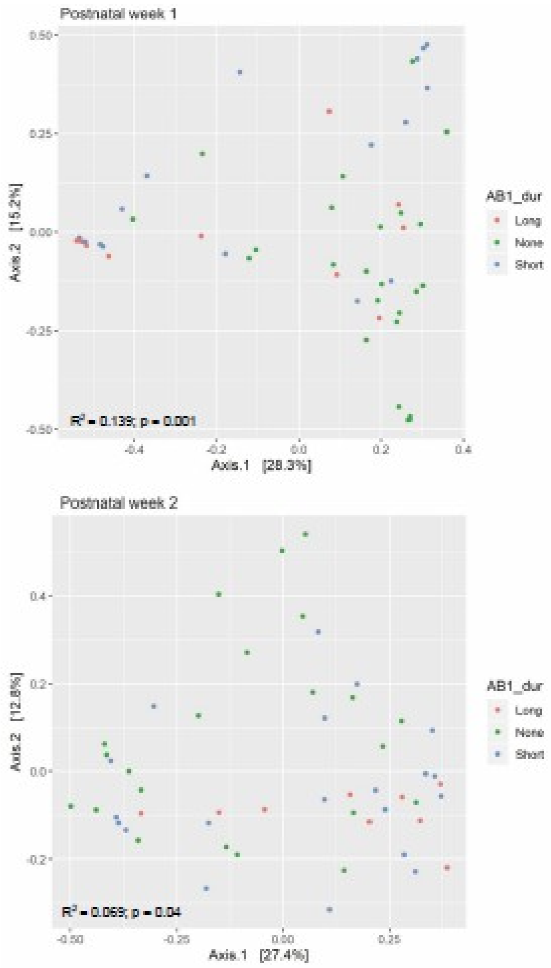
Postnatal week 1
Axis.1 [28.3%]
Axis.2 [15.2%]
AB1_dur
Long
None
Short
R^2 = 0.139; p = 0.001
Postnatal week 2
Axis.1 [27.4%]
Axis.2 [12.8%]
AB1_dur
Long
None
Short
R^2 = 0.069; p = 0.04

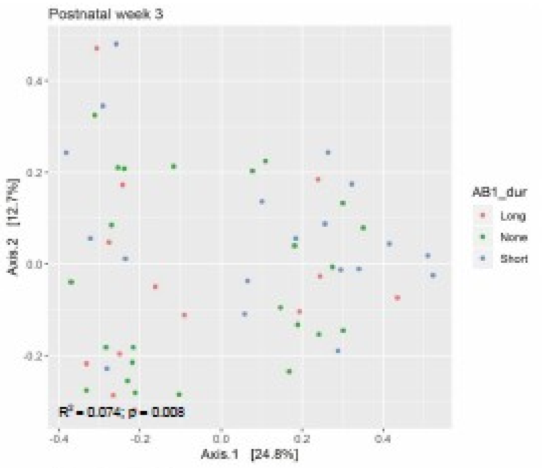
Postnatal week 3
Axis.2 [12.7%]
Axis.1 [24.8%]
AB1_dur
Long
None
Short
R² = 0.074; p = 0.008

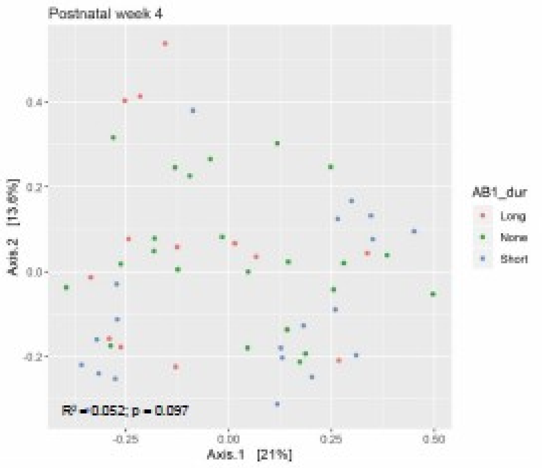
Postnatal week 4
Axis.2 [13.6%]
Axis.1 [21%]
AB1_dur
Long
None
Short
R² = 0.052; p = 0.097

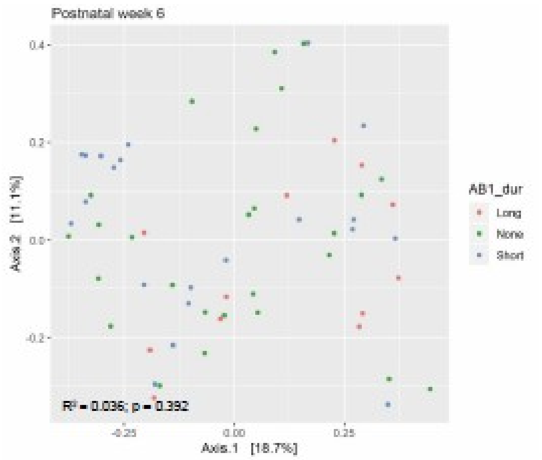
Postnatal week 6
0.4
0.2
0.0
-0.2
Axis.2 [11.1%]
-0.25
0.00
0.25
Axis.1 [18.7%]
R² = 0.036; p = 0.392
AB1_dur
Long
None
Short
