# Supplementary material for: Dynamics of the bacterial gut microbiota in preterm and term infants after intravenous amoxicillin/ceftazidime treatment
Source: BMC Pediatr. 2020 May 7;20:195. doi: 10.1186/s12887-020-02067-z (PMC7204001; doi:10.1186/s12887-020-02067-z)
Supplement: Supplementary file 2 [file 12887_2020_2067_MOESM2_ESM.docx]

Additional file 2. **Clinical factors included during redundancy analysis (RDA).**

Factor	Unit
Gestational age	days
Birthweight	grams
Delivery mode	-
Singlet/twin/triplet	-
Gender	-
Postnatal age	weeks
Maternal antibiotics around birth	yes/no
Maternal antibiotics at least 48 hours after birth	yes/no
Maternal preeclampsia	yes/no
Prolonged rupture of membranes	yes/no
Ventilation	yes/no
Continuous positive airway pressure	yes/no
Days of total parenteral nutrition	days
Days until full enteral feeding	days
Food intolerance	yes/no
Proportion of parenteral/enteral nutrition per week	fraction
Percentage human milk and formula feeding per week	percentage
Average percentage of human milk feeding throughout the first six postnatal weeks	percentage
Antibiotic treatment duration	days
