# Supplementary material for: Dynamics of the bacterial gut microbiota in preterm and term infants after intravenous amoxicillin/ceftazidime treatment
Source: BMC Pediatr. 2020 May 7;20:195. doi: 10.1186/s12887-020-02067-z (PMC7204001; doi:10.1186/s12887-020-02067-z)

Additional file 3. **Redundancy analysis using microbiota composition profiles from (A) control infants and (B) all infants.** Species with a 15-100% fit into the ordination space and explanatory variables that significantly explain variation are shown. Abbreviations: C-section: caesarean section, AB: antibiotics, FEF: full enteral feeding, PNW: postnatal weeks.

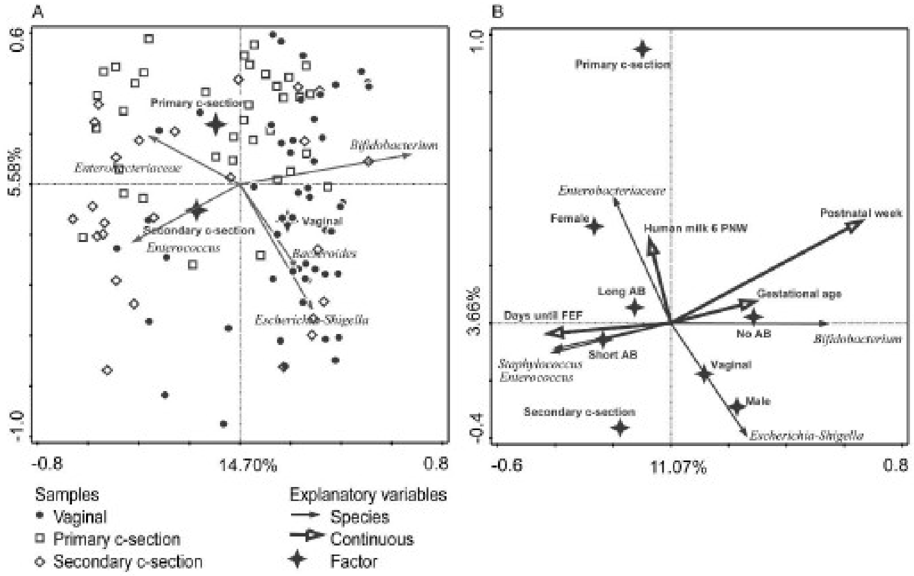

Supplement: Supplementary file 3 [file 12887_2020_2067_MOESM3_ESM.docx]
