# Supplementary material for: Dynamics of the bacterial gut microbiota in preterm and term infants after intravenous amoxicillin/ceftazidime treatment
Source: BMC Pediatr. 2020 May 7;20:195. doi: 10.1186/s12887-020-02067-z (PMC7204001; doi:10.1186/s12887-020-02067-z)

Additional file 4. **Richness and diversity in samples with different dominating bacterial taxa**. Taxa were considered dominant in a sample when it was the most abundant taxon and at least 10% more abundant than the second most abundant taxon. When the difference between the two most abundant taxa was less than 10%, is was considered a mixed community. Boxplots show the median, 25th and 75th percentiles, and minimal and maximal values with the exception of outliers (circles) and extremes (asterisks). Lines above the graph indicate between which communities a significant difference in richness/diversity was observed.

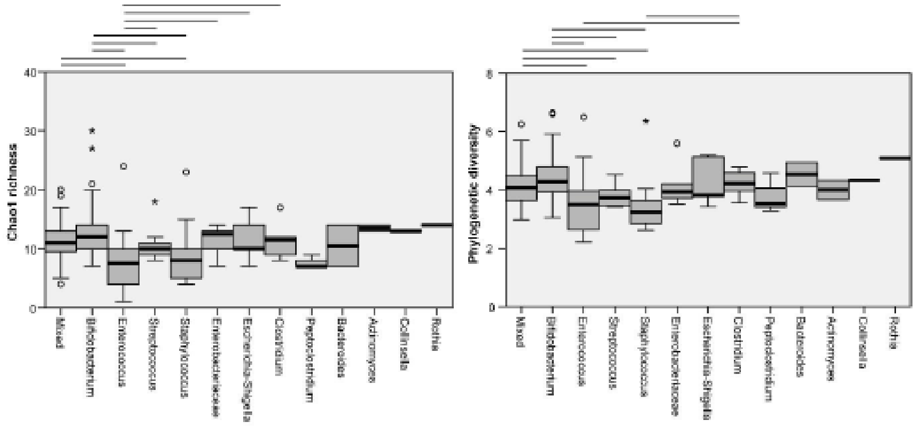

Supplement: Supplementary file 4 [file 12887_2020_2067_MOESM4_ESM.docx]
