# Supplementary material for: Dynamics of the bacterial gut microbiota in preterm and term infants after intravenous amoxicillin/ceftazidime treatment
Source: BMC Pediatr. 2020 May 7;20:195. doi: 10.1186/s12887-020-02067-z (PMC7204001; doi:10.1186/s12887-020-02067-z)

Additional file 5. **Microbiota composition profiles during the first six postnatal weeks in preterm and term infants receiving no, short or long antibiotic treatment.** Average relative abundances per time point are shown.

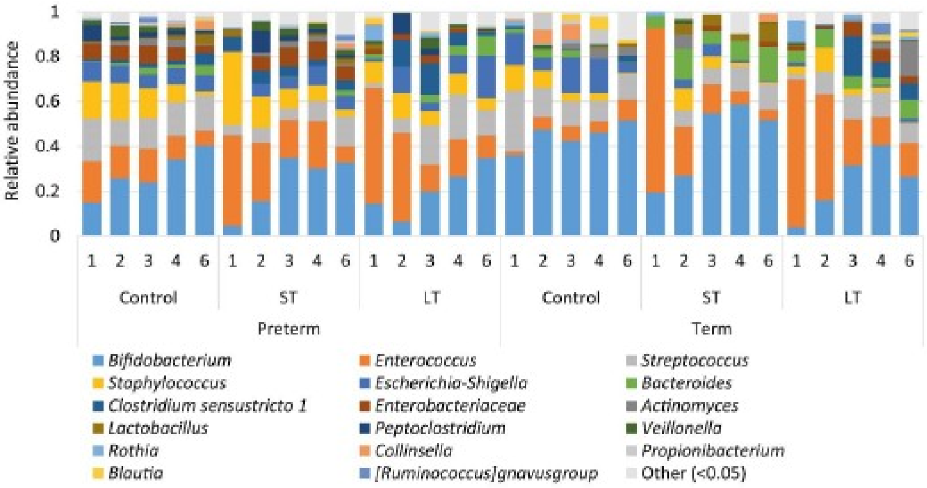

Supplement: Supplementary file 5 [file 12887_2020_2067_MOESM5_ESM.docx]
